# A relation based measure of semantic similarity for Gene Ontology annotations

**DOI:** 10.1186/1471-2105-9-468

**Published:** 2008-11-04

**Authors:** Brendan Sheehan, Aaron Quigley, Benoit Gaudin, Simon Dobson

**Affiliations:** 1 Systems Research Group, School of Computer Science and Informatics, University College Dublin, Belfield, Dublin 4, Ireland

## Abstract

**Background:**

Various measures of semantic similarity of terms in bio-ontologies such as the Gene Ontology (GO) have been used to compare gene products. Such measures of similarity have been used to annotate uncharacterized gene products and group gene products into functional groups. There are various ways to measure semantic similarity, either using the topological structure of the ontology, the instances (gene products) associated with terms or a mixture of both. We focus on an instance level definition of semantic similarity while using the information contained in the ontology, both in the graphical structure of the ontology and the semantics of relations between terms, to provide constraints on our instance level description.

Semantic similarity of terms is extended to annotations by various approaches, either though aggregation operations such as min, max and average or through an extrapolative method. These approaches introduce assumptions about how semantic similarity of terms relates to the semantic similarity of annotations that do not necessarily reflect how terms relate to each other.

**Results:**

We exploit the semantics of relations in the GO to construct an algorithm called SSA that provides the basis of a framework that naturally extends instance based methods of semantic similarity of terms, such as Resnik's measure, to describing annotations and not just terms. Our measure attempts to correctly interpret how terms combine via their relationships in the ontological hierarchy. SSA uses these relationships to identify the most specific common ancestors between terms. We outline the set of cases in which terms can combine and associate partial order constraints with each case that order the specificity of terms. These cases form the basis for the SSA algorithm. The set of associated constraints also provide a set of principles that any improvement on our method should seek to satisfy.

**Conclusion:**

We derive a measure of semantic similarity between annotations that exploits all available information without introducing assumptions about the nature of the ontology or data. We preserve the principles underlying instance based methods of semantic similarity of terms at the annotation level. As a result our measure better describes the information contained in annotations associated with gene products and as a result is better suited to characterizing and classifying gene products through their annotations.

## Background

Although the semantic similarity between two GO terms has been extensively investigated [1-4], how to define similarity between two gene products based on GO annotations for a specific application remains unclear [5]. To date annotation similarity has been computed by four general approaches: the *set-based *approach; the *graph-based *approach; the *vector-based *approach; and the *term-based *approach. In the set-based approach an annotation is viewed as a 'bag of words'. Two annotations are similar if there is a large overlap between their sets of terms. A graph-based approach views similarity as a graph-matching procedure. Vector-based methods embed annotations in a vector space where each possible term in the ontology forms a dimension. Term-based approaches compute similarity between individual terms and then combine these similarities to produce a measure of annotation similarity.

All the above approaches do not consider the semantics of relationships between terms. How terms are related can significantly alter how an annotation, which is a set of terms, is interpreted. In the GO there are two main types of relations: is_a and part_of. The is_a relation represents a taxonomic relationship between terms that can be modeled using the improper subset relation, which is a partial ordering of terms. The part_of relation represents a partonomic relationship between terms that can also be modeled in terms of a partial order. Though the partial orders represented by taxonomies and partonomies are well understood there has been little attention given as to how these two partial orderings combine. Using the various cases identified by combining taxonomies and partonomies we construct an algorithm called SSA (*S*emantic *S*imilarity of *A*nnotations) that identifies the terms that can be associated with an annotation and terms that relate to both annotations. Instances associated with these terms are then used to construct a Resnik-like measure of annotation similarity thus extending the underlying intuitions behind this term-based measure to the annotation level.

A measure of term or annotation similarity should be based on a set of principles that form the basis for what is considered similar. The nature of similarity has been the focus of intense research in the areas of *aesthetics *[6,7] and *psychology *[8]. In mathematics properties such as *identity*, *symmetry *and the *triangle inequality *have been used to form the basis of measures of similarity of mathematical objects. Principles of term and annotation similarity have been suggested by various authors. This work intends to build on these principles and introduce additional principles that a measure of similarity should seek to satisfy. 

Similarity between objects is normally expressed as a number that ranges along an interval on the real numbers ℝ. However the main purpose of similarity is usually to determine whether two or more objects are similar to a reference object. For this reason a measure of similarity can be viewed as a partial order on a set of objects, the actual numbers play only a secondary purpose. For example, we may say that an object *X *is more similar to *Z *than another object *Y*. Formally this is expressed as *sim*(*X, Z*) > *sim*(*Y*, *Z*). 

In the study of ontological similarity Lin [9] develops the principles of *commonality *and *difference *when constructing a measure of *term similarity*. The greater the commonality between objects the greater the similarity. Likewise, the greater the difference between objects the greater the dissimilarity. The source of both the commonality and difference between terms depends on the method chosen to measure the *descriptiveness *of terms. Different sources of descriptiveness may result in different orderings of similarity between terms or annotations.

Popescu *et al*. [10] recognize that an important property of *term similarity *is that two different terms should have a non-zero similarity value if the terms are related. They also recognize that an important property of *annotation similarity *is that the descriptiveness of annotations should be greater than or equal to the descriptiveness of its constituent terms. In this paper this property is called the *monotonicity property*.

In defining a measure of similarity a set of relevant properties that objects can be compared along are identified. In ontological similarity, whether of terms or annotations, there are two main sources of similarity: the *conceptual *or *structural *level; and the *instance *level. At the structural level we may consider such properties as graph distance, graph similarity, relation types, common ancestors, etc. At the instance level we consider the set of instances associated with a term or annotation. Our measure of ontological similarity combines aspects from both levels. Here we survey how various measures of annotation similarity combine these properties in various ways to form the basis for a measure of descriptiveness of a term or annotation.

### Set-Based Approaches

Set based methods for measuring the similarity of annotations are based on the Tversky ratio model of similarity [8,11] which is a general model of distance between sets of terms. It is represented by the formula

$$\frac{f(G_{1}\cap G_{2})}{f(G_{1}\cap G_{2})+\alpha *f(G_{1}-G_{2})+\beta *f(G_{2}-G_{1})}$$

where *G*_1 _and *G*_1 _are sets of terms or annotations from the same ontology and *f *is an additive function on sets (usually set cardinality). For *α *= *β *= 1 we get the Jaccard distance between sets:

$$S_{Jaccard}=\frac{f(G_{1}\cap G_{2})}{f(G_{1}\cup G_{2})}$$

and for *α *= *β *= $\frac{1}{2}$ we get the Dice distance between sets [11]:

$$S_{Dice}=\frac{2*f(G_{1}\cap G_{2})}{f(G_{1})+f(G_{2})}$$

In this situation the source of descriptiveness of an annotation is its set of terms. Each term and its set of associated instances is considered independent of other terms. The commonality and difference between annotations is modeled as set intersection and difference of sets of terms respectively. Set-based approaches return a similarity of zero if they do not share common terms ignoring the fact that terms may be closely related. Because of the atomic nature of terms in the set-based approach the monotonicity property does not apply.

### Vector-Based Approaches

Vector-based methods embed ontological terms in a vector space by associating each term with a dimension. Usually a vector is binary consisting of 0's and 1's where 0 denotes the absence (resp. presence) of a term (along a particular dimension) in an annotation. This has the advantage that standard clustering techniques on vector spaces such as k-means can be applied to group similar terms. What is required is a means of measuring the size of vectors. This can be achieved by embedding terms in a metric space (usually Euclidean). The most common method of measuring similarity between vectors of terms is the cosine similarity

$$s_{v}(G_{1},G_{2})=\frac{v_{1}\cdot v_{2}}{\left|v_{1}||v_{2}\right|}$$

where *v*_*i *_represents a vector of terms constructed from an annotation (group of terms) *G*_*i*_. |·| corresponds to the size of the vector and • corresponds to the dot product between two vectors. The source of descriptiveness, commonality and difference is the same as the situation for set-based approaches.

### Graph-Based Approaches

An ontology is a directed, acyclic graph (DAG) whose edges correspond to relationships between terms. Thus it is natural to compare terms using methods for graph matching and graph similarity. We may consider the similarity between annotations in terms of the sub-graph that connects terms within each annotation. Annotation similarity is then measured in terms of similarity between two graphs. Graph matching has only a weak correlation with similarity between terms. It is also computationally expensive to compute, graph matching being an NP-complete problem on general graphs [12].

The descriptiveness of an annotation is modeled by the set of nodes and edges associated with a subgraph. Commonality between annotations is based on the set intersection while difference is modeled by the set difference where each set consists of the nodes and edges associated with each subgraph. Alternatively, the set of edges may be ignored and only common terms of both graphs are considered [13-15].

### Improving Similarity Measures by Weighting Terms

Set, vector and graph-based methods for measuring similarity between annotations can be improved by introducing a weighting function into the similarity measure. For example, the weighted Jaccard distance can be formulated as:

$$S_{WeightedJaccard}(G_{1},G_{2})=\frac{\sum_{\left\{T_{i}\in G_{1}\cap G_{2}\right\}}m(T_{i})}{\sum_{\left\{T_{j}\in G_{1}\cup G_{2}\right\}}m(T_{j})}$$

where, as before, *G*_1 _and *G*_2 _are annotations or sets of terms describing data (e.g. a gene product), *T*_*x *_is the *x*^*th *^term from a set of terms and *m*(*T*_*x*_) denotes the weight of *T*_*x*_. This weighting function can be used to represent various properties of a term or annotation such as a measure of vagueness, uncertainty, sense of preference or a combination of the above. The vector-based approach may be extended so that values along a particular dimension can lie on the interval [0, 1] or [0, ∞). The graph-based approach can be extended by weighting the edges between terms in the graph.

Assigning a weight to each term in an annotation allows for the possibility of introducing the monotonicity property into a similarity measure. Using the monotonicity property, the weight associated with an annotation should be greater than or equal to the weight associated with any of its constituent terms. Weights can form an additional basis on which to measure the descriptiveness of a term or annotation.

#### Instance-Based Weights

One approach to assigning weight to an ontological term is to measure how *informative *a term is in describing data. A method of measuring information is to analyze a term's use in a corpus against the general use of ontological terms in the same corpus. Information is measured using the *surprisal *function:

(1) *IC*_*Corpus*_(*T*_*i*_) = -log(*p*(*T*_*i*_))

where *p*(*T*_*i*_) corresponds to the probability of a term *T*_*i *_or its taxonomic descendants occurring in a corpus. For example, consider the case where there are 30 distinct instances in a corpus and 5, 3 and 2 of these instances are annotated by the terms *T*_*i*_, *T*_*j *_and *T*_*k *_respectively. If *T*_*j *_and *T*_*k *_are sub-types or children of *T*_*i *_and do not have child terms themselves then $IC_{Corpus}(T_{i})=-\log\left(\frac{5+3+2}{30}\right)\approx 1.099$.

#### Other Weighting Approaches

Other measures of information can be used not necessarily relying on corpus data. One measure [16] relies on the assumption that how the ontology is constructed is semantically meaningful:

$$IC_{Ont}(T_{i})=1-\frac{\log(desc(T_{i})+1)}{\log(numTerms)}$$

where *desc*(*T*_*i*_) returns the number of descendants of term *T*_*i *_and *numTerms *refers to the total number of terms in the ontology.

### Term-Based Approaches

In term-based approaches similarity between pairs of terms from each annotation are computed. These weightings are then combined in order to characterize the similarity between annotations as a whole. There are several ways to combine similarities of pairs of terms such as the min, max or average operations. Term-based approaches depend on a function *s*(*T*_*i*_, *T*_*j*_) where *T*_*i *_and *T*_*j *_are terms from two annotations *G*_1 _and *G*_2 _respectively. *s*(*T*_*i*_, *T*_*j*_) provides a measure of distance/similarity between these two terms. Once distances has been measured between all possible pairs of terms they are then aggregated using an operation such as max or the average of all distances. For example:

$$S_{avg}(G_{1},G_{2})=\frac{\sum_{i=1}^{n}\sum_{j=1}^{m}s(T_{i},T_{j})}{m*n}$$

More sophisticated term based approaches combine multiple measures of term similarity and aggregate similarity values using more complex functions, for example [17].

#### Graphical Measures of Term Similarity

**Figure 1:**
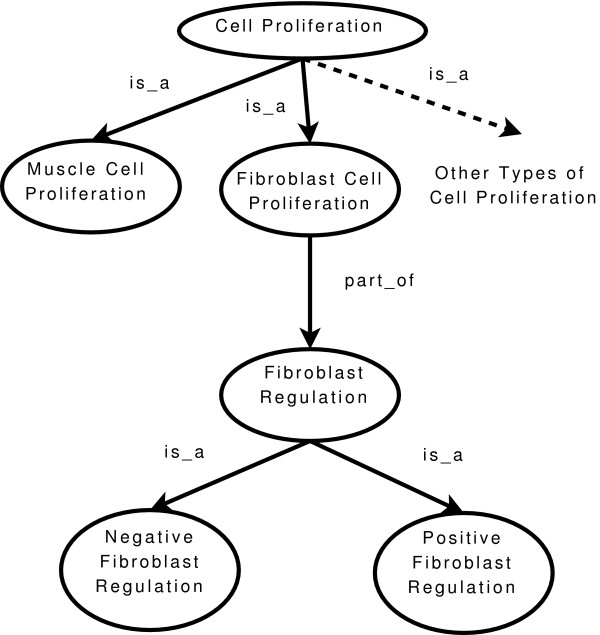
**An Example of an Ontology of GO Terms**. Nodes in the graph correspond to ontological terms. Edges correspond to relations between terms. Lower down terms in the diagram are descendants of terms higher up in the diagram if connected by an edge.

The simplest approach to measuring similarity between ontological terms using the graph structure is to measure the shortest path distance between terms in the graph [18,19]. Referring to figure 1, in terms of graph distance, we may consider the terms 'muscle cell proliferation' and 'fibroblast cell proliferation' (graph distance of 2) as being more similar than the former term with 'fibroblast regulation' (graph distance of 3). However the graph distance has only a weak correlation with similarity of terms. The semantic similarity between 'positive fibroblast regulation' and 'negative fibroblast regulation' is far greater than the similarity between 'muscle cell proliferation' and 'fibroblast cell proliferation' even though both examples have a graph distance of two. A simple graph distance-based measure of similarity does not model in a consistent way any notion of commonality or difference between terms.

A more refined use of graph distance as a basis for a measure of term similarity is found in the *Wu-Palmer *measure of similarity [20]. It uses the idea that the distance from the root to the *lowest common taxonomic ancestor *(LCTA) measures the commonality between two terms while the sum of the distance between the LCTA and each term measures the difference between two terms. Combining these aspects results in the formula:

$$s_{Wu-Palmer}(T_{1},T_{2})=\frac{2*dist(T_{lcta},T_{root})}{dist(T_{1},T_{lcta})+dist(T_{2},T_{lcta})+2*dist(T_{lcta},T_{root})}$$

Where *T*_1 _and *T*_2 _are the two terms being compared, *T*_*lcta *_is the term that corresponds to the lowest common taxonomic ancestor between *T*_1 _and *T*_2_. *T*_*root *_denotes to root node of the ontology (assuming that the ontology has only one root). *dist*(*T*_*i*_, *T*_*j*_) denotes the graph distance between terms *T*_*i *_and *T*_*j*_. The 2 * *dist*(*T*_*lcta*_, *T*_*root*_) component of the denominator serves to normalize the measure.

#### Instance-Based Measures of Term Similarity

Similarity may be measured using an instance based measure of semantic similarity as computed by either Resnik (eqn. 2) or Lin (eqn. 3). Resnik [21,22] exploits the informativeness of the lowest common ancestor between terms as a measure of semantic similarity:

(2) *s*_*Resnik*_(*T*_*i*_, *T*_*j*_) = *IC*_*Corpus*_(*T*_*lcta*_)

where *T*_*lcta *_denotes the lowest common taxonomic ancestor between ontological terms *T*_*i *_and *T*_*j*_. This measure only accounts for the commonality between terms.

Another method of measuring similarity derived by Lin [9] is:

(3) $$s_{Lin}(T_{i},T_{j})=\frac{2*IC_{Corpus}(T_{lcta})}{IC_{Corpus}(T_{i})+IC_{Corpus}(T_{j})}$$

which has the advantage that it maps onto values on the interval [0, 1] unlike Resnik's measure which maps onto the interval [0, ∞). Lin's measure also accounts for both the commonality and difference between terms. Resnik's measure does have the desirable property that terms close to the root of the ontology have a low similarity however. This is not the case for Lin's measure.

The only structural property that both Resnik and Lin exploit is the lowest common taxonomic ancestor. To overcome this weakness Jiang and Conrath [23] integrate graph distance based measures of similarity into information based approaches. They construct a generalized weighting measure between a child and its immediate parent that accounts for the number of out edges and depth of terms along the shortest path between the compared terms in the ontology. While they acknowledge that other relation types might be relevant to measuring similarity their measure is based solely on the taxonomic or is_a relations in the ontology.

### New Approaches to Annotation Similarity

Beyond the set, vector, graph and term-based approaches to measuring similarity of annotations exist other methods that introduce the additional properties discussed above such as *monotonicity *and taking into account the semantics of ontological relations.

#### Similarity Based on Fuzzy Measures

The monotonicity property leads naturally to the use of fuzzy measures as a basis for measuring the descriptiveness of an annotation. Using the information content measure of terms described in eqn. 1 as the basis for measuring similarity a fuzzy measure is constructed. A fuzzy measure is a weighting on sets of terms such that the weight associated with a set of terms is greater than or equal to the weight associated with any of its subsets.

Popescu *et al*. [10] use fuzzy measures to induce a weighting *m *for an annotation from its constituent terms. This weight is extrapolated from the weights of individual terms by using the formula for constructing a Sugeno *λ*-fuzzy measure: For a set of terms *G*_*a*_, *G*_*b *_and *G*_*c *_where *G*_*c *_= *G*_*a *_∪ *G*_*b *_and *G*_*a *_∩ *G*_*b *_= ∅ a *λ*-fuzzy measure for *G*_*c *_is

*m*_*λ*_(*G*_*c*_) = *m*_*λ*_(*G*_*a*_) + *m*_*λ*_(*G*_*b*_) + λ * *m*_*λ*_(*G*_*a*_) * *m*_*λ*_(*G*_*b*_)

where *λ *is a value that ensures that *m*(*G*_*c*_) ≥ *m*(*G*_*a*_) and *m*(*G*_*c*_) ≥ *m*(*G*_*b*_). Given that the weights (fuzzy measure densities) *m *for individual terms *T*_*i *_in an annotation are known then *λ *can be determined by solving the following equation:

$$1+\lambda =\prod_{T_{i}}\left(1+\lambda m(T_{i})\right)$$

In [10] the weight for each term is based on the ICCorpus measure (eqn. 1). The similarity of two annotations, represented by a set of terms *G*_1 _and *G*_1 _from the same ontology, are compared using the similarity function:

$$S_{FMS}(G_{1},G_{2})=\frac{m_{G_{1}}(G_{1}\cap G_{2})+m_{G_{2}}(G_{1}\cap G_{2})}{2}$$

where $m_{G_{1}}$ and $m_{G_{2}}$ are the *λ*-fuzzy measure functions that characterize *G*_1 _and *G*_2 _respectively. The relatedness of terms is accounted for by augmenting each annotation with the lowest common ancestors for each pair of terms from each annotation. This ensures a non-zero similarity between annotations containing related terms.

However, an ontology models other aspects of relatedness that should be taken into account. Relations between terms in an annotation can be used to identify redundant terms whose relevance to the descriptiveness of an annotation is already accounted for by other terms. For example, if two terms in an annotation are taxonomically related the existence of the parent term is implied by the existence of the child term.

If redundancy of terms is not taken into account it may lead to too many or too few instances being associated with the term. This is especially true when a term is part_of another term. The instances associated with the annotation consist of the parts and not what the instances are part of.

#### Exploiting Semantics of Ontological Relations

Wang *et al*. [14] account for the different contributions that terms related by is_a and part_of relations make to the descriptiveness of a term. The semantic contribution that ancestor terms make to a child term is calculated by:

$$SV(T_{i})=\sum_{T_{j}\in T_{anc,i}}s_{T_{i}}(T_{j})$$

where *T*_*anc*, *i *_denotes the ancestors of term *T*_*i *_and $s_{T_{i}}$ is calculated as

$$\{\begin{array}{l} s_{T_{i}}(T_{i})=1\\ s_{T_{i}}(T_{j})=\max\{w_{e}*s_{T_{i}}(T_{k})\\ |T_{k}\in \text{childrenOf}(T_{j})\}\text{ if }T_{j}\neq T_{i} \end{array}$$

where *w*_*e *_∈ [0, 1] is a number that corresponds to the semantic contribution factor for edge *e*. *childrenOf*(*T*_*x*_) is a function that returns the immediate children of *T*_*x *_that are ancestor terms of *T*_*i*_. In this paper *w*_is_a _= 0.8 and *w*_part_of _= 0.6. The similarity of two terms is computed by the formula

$$s(T_{i},T_{j})=\frac{\sum_{T_{k}\in T_{anc,i}\cap T_{anc,j}}\left(s_{T_{i}}(T_{k})+s_{T_{j}}(T_{k})\right)}{SV(T_{i})+SV(T_{j})}$$

A term-based approach is taken to measuring the similarity between annotations *G*_1 _and *G*_2_. The similarities of the most similar pairs of terms from each annotation are averaged over to calculate the similarity between annotations:

$$S_{Wang}(G_{1},G_{2})=\frac{\sum_{T_{i}\in G_{1}}s(T_{i},G_{2})+\sum_{T_{j}\in G_{2}}s(T_{j},G_{1})}{\left|G_{1}\right|+\left|G_{2}\right|}$$

where $s(T_{x},G_{y})=\max_{T_{y}\in G_{y}}(s(T_{x},T_{y}))$ and |*G*_*y*_| denotes the number of terms in annotation *G*_*y*_.

Wang *et al*. make the observation that the instance based measures of term similarity will produce varying results based on the corpus chosen. They keep a fixed value for the contribution each relation type makes to the descriptiveness of a term. This does not account for the varying influence of terms on each other throughout the ontology even if the graph distance is the same. Exploiting the corpus statistics, if used appropriately, may account for this drawback. As with all term-based methods, where terms from each annotation are compared in a pairwise fashion, it is difficult to see how the monotonicity property is ensured when measuring the similarities between two annotations.

## Methods

The Gene Ontology relates terms using is_a and part_of relations. We develop a measure of informativeness that provides a description of an annotation that takes into consideration the relations between terms. We use the informativeness measure of a term (eqn. 1) as the basis for providing a description of an annotation. We define an algorithm called *SSA *that combines the instances of terms while taking into account how these sets of instances are related by how their associated terms are related in the ontology. This results in a set of instances that can be said to be associated with an annotation and not just a term. We can then extend the concept of instance based semantic similarity of terms, such as Resnik's measure, to annotations.

### Interpreting Annotations from Taxonomies

A taxonomy induces a partial ordering on a set of terms by the improper subset relation ⊆. If *T*_*i *_is_a *T*_*k *_and *T*_*j *_is_a *T*_*k *_then the set of instances associated with both *T*_*i *_and *T*_*j *_are subsets of *T*_*k*_. Assuming that we know of all possible instances that can be associated with a term, whatever properties that instances of both *T*_*i *_and *T*_*j *_share can be associated with any of the instances that can be associated with *T*_*k*_. This forms the basis for measuring the commonality between terms used in instance-based measures of similarity between terms.

The difference between terms *T*_*i *_and *T*_*j *_is modeled by the difference between the set of instances associated with each term. If we have two or more terms from a taxonomy in an annotation then it is reasonable to argue that the set of instances associated with an annotation should be the intersection of the set of instances associated with each term. The informativeness of the annotation is then based on the set of instances resulting from this intersection.

### Interpreting Annotations from Partonomies

The part_of relation between terms denotes the concept that one term is '*part of *' another. It provides an alternative notion of relatedness between terms. An ontology consisting only of part_of relations is known as a *partonomy*. An example of a simple partonomy is *wheel *part_of *car*. It would not make sense to say that a *wheel *is_a *car*. The study of partness is complicated by the fact that there are many kinds of part_of relations. Yet the study of partness, known as *mereology *[24], has shown that there are also common aspects to all types of part_of relations, namely that part_of relations form a partial ordering on the sets of instances associated with each term.

According to the GO Consortium's usage guidelines since 2004 [25] the part_of relation should be interpreted as 'necessarily part of' where *T*_*i *_part_of *T*_*j *_means that all instances of *T*_*i *_are part of one or more instances of *T*_*j*_. The converse is not necessarily true. For example, all nuclei are part of cells but not all cells contain a nucleus. Bittner [26] models such a part_of relation using an improper partial order i.e. for term *T*_*i *_with descendant terms *T*_*j*_.

(4) *T*_*j *_≤_*part_of *_*T*_*i *_∀*T*_*j *_part_of *T*_*i*_

Annotations consisting of terms such that one term is part_of another should view the child term as being relevant to the annotation while the parent term provides redundant, contextual information. For example, consider an annotation consisting of two terms *T*_*i *_and *T*_*j *_from a partonomy. If *T*_*j *_part_of *T*_*i *_then the annotation should be interpreted as *the set of instances of T*_*j*_. All we can say is that the number of instances of *T*_*i *_associated with the annotation can be no more than the number of instances of *T*_*j*_. In general, an annotation consisting of terms belonging to a partonomy consists of terms that provide the set of instances that can be associated with the annotation while other terms provide the context in which these instances are embedded.

### Partial Order Constraints for GO Annotations

**Figure 2:**
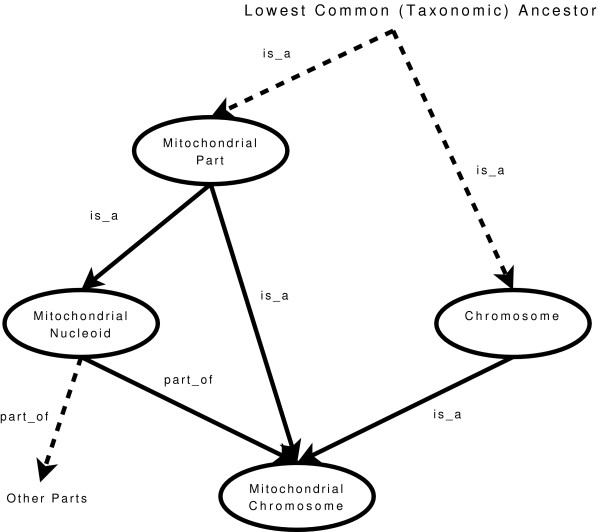
**A Subset of GO Terms and Relations**. An example of where the part_of relation plays an important role in interpreting annotations. If an annotation contains the term 'mitochondrial chromosome' then all other terms shown in the graph are redundant. The diagram also shows various cases that describe how terms relate to each other.

Figure 2 shows a subset of the GO consisting of both part_of and is_a relations. According to the taxonomic is_a relations both 'mitochondrial chromosome' and 'mitochondrial nucleoid ' are 'mitochondrial part's. A measure of descriptiveness of a term should at least say that both 'mitochondrial chromosome' (a) and 'mitochondrial nucleoid ' (b) are more descriptive than 'mitochondrial part' (c), i.e. *a*, *b *⊆ *c*. Likewise, the part_of relation in figure 2 indicates that *a *≤_*part_of *_*b*. Here we can see how the part_of relation provides additional indirect information about descriptiveness not represented by the taxonomic relations. If an annotation consists of the terms 'mitochondrial chromosome' and 'mitochondrial nucleoid' then the annotation should be interpreted as *the set of instances of 'mitochondrial chromosome'*. If the terms 'mitochondrial part' and 'chromosome' are added to the annotation then the same set of instances should be associated with the annotation. All additional terms are already implied by the existence of 'mitochondrial chromosome' in the annotation. If we had either treated the part_of relation as an is_a relation or ignored it then the annotation would have been interpreted as *the set of instances that are both 'mitochondrial chromosome' and 'mitochondrial nucleoid'*. With this interpretation we would have possibly returned an empty set of instances since chromosomes are not nucleoids.

The GO consists of many examples similar to the one described above. In general, the GO can be viewed as a taxonomy interspersed with part_of relations. Two terms are said to be *directly related *if there exists a series of relations on a single path between them. Terms that are not directly related along a path in the graph are *indirectly related *via a common ancestor. For example there may be other terms that are part_of 'mitochondrial nucleoid' in which case the term 'mitochondrial chromosome' is only related to the other parts by an indirect path of part_of relations. Though not shown, the terms 'mitochondrial nucleoid' and 'chromosome' are only indirectly related via a common ancestor through a number of is_a relations. When interpreting an annotation it is necessary to account for such situations.

In general, as described in table 1, there are nine cases to handle when trying to account for how terms are related. Terms or their taxonomic descendants may be directly related to each other in the ontology via a single path. Alternatively they may be indirectly related to each other via a common ancestor in which case we consider the two paths from the common ancestor to each term. A path may be homogeneous in that it consists of relations of only one type i.e. all relations are either only is_a or only part_of. Such paths are denoted by IS and PART respectively. A path that is inhomogeneous, consisting of both is_a and part_of relations, is denoted by MIXED.

**Table 1:** Partial Order Constraints

	Situation	Ordering
Directly*	*T*_*i *_IS *T*_*j*_	*ρ*(*T*_*i*_) ≤ *ρ*(*T*_*j*_)
	*T*_*i *_PART *T*_*j*_	*ρ*(*T*_*i*_) ≤ *ρ*(*T*_*j*_)
	*T*_*i *_MIXED *T*_*j *_via *T*_*k*_	*ρ*(*T*_*i*_) ≤ *ρ*(*T*_*k*_) ≤ *ρ*(*T*_*j*_)

Indirectly Via *T*_lca _*	*T*_*i *_IS *T*_*lca*_, *T*_*j *_IS *T*_*lca*_	*ρ*(*T*_*i*_), *ρ*(*T*_*j*_) ≤ *ρ*(*T*_*lca*_)
	*T*_*i *_PART *T*_*lca*_, *T*_*j *_IS *T*_*lca*_	*ρ*(*T*_*i*_) ≤ *ρ*(*T*_*j*_) ≤ *ρ*(*T*_*lca*_)
	*T*_*i *_PART *T*_*lca*_, *T*_*j *_PART *T*_*lca*_	*ρ*(*T*_*i*_), *ρ*(*T*_*j*_) ≤ *ρ*(*T*_*lca*_)
	*T*_*i *_MIXED *T*_*lca *_via *T*_*k*_, *T*_*j *_IS *T*_*lca*_	(*ρ*(*T*_*i*_) ≤ *ρ*(*T*_*k*_)), *ρ*(*T*_*j*_) ≤ *ρ*(*T*_*lca*_)
	*T*_*i *_MIXED *T*_*lca *_via *T*_*k*_, *T*_*j *_PART *T*_*lca*_	*ρ*(*T*_*i*_), *ρ*(*T*_*j*_) ≤ *ρ*(*T*_*k*_) ≤ *ρ*(*T*_*lca*_)
	*T*_*i *_MIXED *T*_*lca *_via *T*_*k*_, *T*_*j *_MIXED *T*_*lca *_via *T*_*m*_	(*ρ*(*T*_*i*_) ≤ *ρ*(*T*_*k*_)), (*ρ*(*T*_*j*_) ≤ *ρ*(*T*_*m*_)) ≤ *ρ*(*T*_*lca*_)

Overview of general forms of relation based ordering for directly and indirectly related terms. Terms are indirectly related via a common ancestor term *T*_*lca*_. Instances of terms *T*_*i *_and *T*_*j *_may be part of the common ancestor *T*_*lca *_via terms *T*_*k *_and *T*_*m *_respectively. *ρ *denotes a function that measures the number of instances (our source of descriptiveness) of terms. These orderings assume complete knowledge of all instances associated with a term.

#### Directly Related Cases

There are three cases to handle when there exists a single path between terms in the ontology: IS, PART and MIXED paths. The first case is the generalized case of taxonomic relations where *T*_*i *_IS *T*_*j*_. For two terms *T*_*i *_and *T*_*j*_, where *T*_*j *_is the parent term and *T*_*i *_is a descendant, and a set of *n *intermediate terms {*T*^*n*^} such that:

$$T_{i}\subseteq T_{1}^{n}\subseteq T_{2}^{n}\ldots \subseteq T_{n-1}^{n}\subseteq T_{n}^{n}\subseteq T_{j}$$

it can be inferred that *T*_*i *_⊆ *T*_*j*_. Where terms are related by a PART path a similar argument can be inferred for how two terms are ordered.

For the MIXED case there exists a mixture of is_a and part_of relations. The nature of the MIXED relationship is ultimately determined by the part_of relations. For example, if *T*_*i *_MIXED *T*_*j *_then this can be interpreted as *T*_*i *_part_of *T*_*j*_. There may be several is_a relations traversed along a MIXED path from *T*_*j *_to *T*_*i *_before a part_of relation is encountered. This means that *T*_*i *_can only be part_of a subset of the instances of *T*_*j*_. This subset is identified by the set of instances associated with the term (labeled *T*_*k *_in table 1) which is the parent term of the first part_of relation encountered along a MIXED path from *T*_*j *_to *T*_*i*_. This results in the partial order:

*T*_*i *_≤ *T*_*k *_≤ *T*_*j*_

where *T*_*i *_is the descendant of *T*_*j*_, *T*_*i *_is the parent and *T*_*k *_denotes the first term before a part_of relation is encountered while traversing the MIXED path in the ontology from *T*_*j *_to *T*_*i*_. This form of reasoning can be further extended along the rest of the MIXED path to produce a more detailed partial order. However if the ultimate goal is to only determine the partial order between *T*_*i *_and *T*_*j *_then such induction of this reasoning is unnecessary.

#### Indirectly Related Homogeneous Cases

There are three cases to handle where both the paths to the common ancestor between terms are homogeneous: IS – IS, PART – PART and IS – PART (or PART – IS). In the first case, where *T*_*i *_IS *T*_*lca *_and *T*_*j *_IS *T*_*lca*_, since both terms *T*_*i *_and *T*_*j *_are taxonomic descendants of a lowest common ancestor *T*_*lca *_then it should be expected that the number of instances associated with *T*_*i *_and *T*_*j *_are less than the number of instances associated with *T*_*lca*. _This results in the partial order

*T*_*i*_, *T*_*j *_≤ *T*_*lca*_

An annotation consisting of two such related terms can be interpreted as *the set of instances that are associated with both T*_*i *_*and T*_*j*_. A similar form of reasoning can be applied to the PART – PART case. The partial order for the final case IS – PART (or PART – IS) can be derived in a similar fashion to the inhomogeneous direct MIXED case. If *T*_*i *_IS *T*_*lca *_and *T*_*j *_PART *T*_*lca *_then it can be inferred that *T*_*j *_PART *T*_*i*_. If an annotation consists of two such terms then it should be interpreted as *the set of instances of T*_*j*_. As a partial order constraint this can be modeled as

*T*_*j *_≤ *T*_*i *_≤ *T*_*lca*_

#### Indirectly Related Inhomogeneous Cases

Indirectly related inhomogeneous cases occur when terms are related by a common ancestor in the ontology and one or both of the paths connecting the common ancestor with each term consists of an inhomogeneous set of relation types. There are three such cases to account for: IS – MIXED (or MIXED – IS), PART – MIXED (or MIXED – PART) and MIXED – MIXED.

The partial order for the first case IS – MIXED (or MIXED – IS) can be handled by considering each path separately. The partial order for the *T*_*i *_IS *T*_*lca *_path is *T*_*i *_≤ *T*_*lca*_. The partial order for the MIXED path is *T*_*j *_≤ *T*_*k *_≤ *T*_*lca *_which is derived in the same way as the directly related MIXED case. Combining the two partial orders results in

(*T*_*j *_≤ *T*_*k*_), *T*_*i *_≤ *T*_*lca*_

If an annotation consists of two such terms then it should be interpreted as *the set of instances of T*_*j *_*that are part of instances that are of type T*_*i *_*and T*_*k*_.

The PART – MIXED (or MIXED – PART) case requires slightly more reasoning about to construct its associated partial order. If *T*_*i *_PART *T*_*lca *_and *T*_*j *_MIXED *T*_*lca *_then it can be inferred that both *T*_*i *_and *T*_*j *_are part of *T*_*lca*_. Because *T*_*j *_is only part of a subset of the instances associated with *T*_*lca*_, the instances associated with *T*_*k*_, then *T*_*i *_can only be part of the set of instances associated with *T*_*k *_also. This results in the partial order

*T*_*j*_, *T*_*i *_≤ *T*_*k *_≤ *T*_*lca*_

An annotation consisting of two such related terms should be interpreted as *the set of instances of T*_*i *_*and T*_*j *_*that are part of the same instances of T*_*k*_.

The final case MIXED – MIXED occurs when paths from both terms to the common ancestor consist of a mixture of relation types. The partial order for such a case can be constructed by looking at each path separately. If *T*_*i *_MIXED *T*_*lca *_then the partial ordering is *T*_*i *_≤ *T*_*k *_≤ *T*_*lca*_. Similarly for *T*_*j *_MIXED *T*_*lca *_we get *T*_*j *_≤ *T*_*m *_≤ *T*_*lca*_. Combining the two partial orders results in

(*T*_*i *_≤ *T*_*k*_), (*T*_*j *_≤ *T*_*m*_) ≤ *T*_*lca*_

If an annotation consists of two such terms then it should be interpreted as *the set of instances of T*_*i*_*and T*_*j*_*that are part of the same instances of T*_*k*_*and T*_*m*_.

### The SSA Algorithm

The SSA algorithm is based on the nine cases of term relatedness described above. The SSA algorithm derives the set of instances that can be associated with an annotation from the set of instances associated with that annotation's constituent terms. There are two aspects to the algorithm: identifying which terms are the contextual, redundant instances and which terms' instances can be associated with the annotation. For example, a contextual instance may be 'mitochondrial nucleoid' that provides the context for the set of instances of 'chromosome'. Throughout we denote the set of contextual terms by *exclTerms *and the set of terms whose instances can be associated with the annotation as *inclTerms*. *numInst(T*_*i*_) denotes the number of instances associated with *T*_*i*_.

The above partial order constraints were constructed under the ideal assumptions assumed by the partial orderings in taxonomies and partonomies. In reality there only ever exists an incomplete set of instances associated with terms and some adjustment of the number of instances is required if the partial order constraints are to be satisfied. Terms that are taxonomically related are guaranteed to satisfy the taxonomic constraints. However, terms that are partonomically related may not satisfy their associated partial order constraints. In these cases some adjustment of the number of instances associated with a term is necessary. For example, if *T*_*i *_PART *T*_*j *_and there are no instances associated with *T*_*j *_in the corpus while there are a number of instances associated with *T*_*i *_then in order to satisfy the PART constraint the number of instances of *T*_*j *_is set equal to the number of instances associated with *T*_*i*_.

The algorithm consists of the following steps:

• For each distinct ordered pair (*T*_*i*_, *T*_*j*_) of terms in annotations *G*_1 _and *G*_2 _respectively

- Identify the case that corresponds to how *T*_*i *_is related to *T*_*j*_

* Terms are assigned to *inclTerms *or *exclTerms *depending on case

* The number of instances associated with a term may be adjusted if the case allows

• Remove any terms from *inclTerms *also found in *exclTerms*

• Return the sets *inclTerms *and *exclTerms*

where an ordered pair of terms (*T*_*i*_, *T*_*j*_) means that (*T*_*i*_, *T*_*j*_) ≠ (*T*_*j*_, *T*_*i*_). In the following sections we identify how each case assigns terms to *inclTerms *and *exclTerms *and adjusts the number of instances associated with each term used to compare annotations.

#### Direct Cases

The IS constraint where one term in an annotation is a special case of another term can be implemented as follows:

**1 **if (*T*_*i *_IS *T*_*j*_)

inclTerms ← inclTerms ∪ *T*_*i*_

exclTerms ← exclTerms ∪ *T*_*j*_

In this situation the term *T*_*j *_is viewed as being the common taxonomic ancestor of both terms.

The PART constraint where one term is a part of another term can be implemented as:

**2 **if (*T*_*i *_PART *T*_*j*_)

inclTerms ← inclTerms ∪ *T*_*i*_

exclTerms ← exclTerms ∪ *T*_*j*_

if (numInst (*T*_*j*_) < numInst(*T*_*i*_))

numInst(*T*_*j*_) = numInst(*T*_*i*_)

In this situation the term *T*_*j *_is viewed as providing the context that instances of *T*_*i *_are part of.

The case is similar for *T*_*i *_MIXED *T*_*j*_. In these cases we are relating terms that belong to two different lines of taxonomic inheritance where terms have a possibly incomplete set of associated instances. In order to ensure that the partial order constraint associated with this case is implemented correctly if *T*_*j *_has fewer instances associated with it than *T*_*i *_then we adjust the number of instances associated with *T*_*j *_to be equal to the number of instances associated with *T*_*i*_.

The MIXED constraint where *T*_*i *_is a part of another term *T*_*j *_via an intermediate term *T*_*k *_can be implemented similarly to the PART case:

**3 **if (*T*_*i *_MIXED *T*_*j*_)

inclTerms ← inclTerms ∪ *T*_*i*_

exclTerms ← exclTerms ∪ *T*_*j*_

exclTerms ← exclTerms ∪ *T*_*k*_

if (numInst(*T*_*k*_) < numInst(*T*_*i*_))

numInst(*T*_*k*_) = numInst(*T*_*i*_)

if (numInst(*T*_*j*_) < numInst(*T*_*i*_))

numInst(*T*_*j*_) = numInst(*T*_*i*_)

In this situation the term *T*_*k *_is viewed as providing the context that instances of *T*_*i *_are part of.

#### Indirect Homogeneous Cases

In the indirect homogeneous cases compared terms *T*_*i *_and *T*_*j *_are indirectly related via a common ancestor *T*_*lca *_along homogeneous paths. The first such case is where *T*_*i *_IS *T*_*lca *_and *T*_*j *_IS *T*_*lca*. _In this situation the number of instances associated with *T*_*lca *_provides a measure of similarity between *T*_*i *_and *T*_*j*_:

**4 **if (*T*_*i *_IS *T*_*lca *_&*T*_*j *_IS *T*_*lca*_)

numInst(*T*_*i*_), numInst(*T*_*j*_) ← min(numInst(*T*_*i*_), numInst(*T*_*j*_))

inclTerms ← inclTerms ∪ *T*_*j *_∪ *T*_*i*_

exclTerms ← exclTerms ∪ *T*_*lca*_

In the case where *T*_*i *_PART *T*_*lca *_and *T*_*j *_PART *T*_*lca*_*T*_*lca *_provides the context in which instances of *T*_*i *_and *T*_*j *_are embedded.

**5 **if (*T*_*i *_PART *T*_*lca *_&*T*_*j *_PART *T*_*lca*_)

numInst(*T*_*i*_), numInst(*T*_*j*_) ← min(numInst(*T*_*i*_) ∩ numInst(*T*_*j*_))

inclTerms ← inclTerms ∪ *T*_*j *_∪ *T*_*i*_

exclTerms ← exclTerms ∪ *T*_*lca*_

if (numInst(*T*_*lca*_) < numInst(*T*_*i*_))

numInst(*T*_*lca*_) = numInst(*T*_*i*_)

Since terms from two different lines of taxonomic inheritance are being compared and the set of instances associated with each term is incomplete an adjustment of the number of instances associated with each term is necessary.

The final homogeneous indirect case occurs when *T*_*i *_PART *T*_*lca *_and *T*_*j *_IS *T*_*lca*_. This is equivalent to *T*_*i *_PART *T*_*j *_since if *T*_*i *_is a part of *T*_*lca *_and *T*_*j *_is a kind of *T*_*lca *_then *T*_*i *_is a part of *T*_*j*_.

**6 **else if (*T*_*i *_PART *T*_*lca *_&*T*_*j *_IS *T*_*lca*_)

inclTerms ← inclTerms ∪ *T*_*i*_

exclTerms ← exclTerms ∪ *T*_*j*_

exclTerms ← exclTerms ∪ *T*_*lca*_

if (numInst(*T*_*j*_) < numInst(*T*_*i*_))

numInst(*T*_*j*_) = numInst(*T*_*i*_)

if (numInst(*T*_*lca*_) < numInst(*T*_*i*_))

numInst(*T*_*lca*_) = numInst(*T*_*i*_)

As with other cases the number of instances associated with each term are adjusted to ensure that the partial order constraint associated with the case is satisfied.

#### Indirect Inhomogeneous Cases

In these cases one or both paths from *T*_*lca *_to terms *T*_*i *_and *T*_*j *_contain inhomogeneous types of relations. Throughout this section the term *T*_*k *_is a term in the ontology such that *T*_*m *_MIXED *T*_*k *_and *T*_*k *_IS *T*_*n *_if *T*_*n *_is an ancestor of *T*_*m *_in the ontology.

The first such case occurs where for two indirectly related terms being compared, *T*_*i *_and *T*_*j*_, there exists an MIXED path from *T*_*i *_to *T*_*lca *_via *T*_*k *_and an IS path from *T*_*j *_to *T*_*lca*_.

**7 **if (*T*_*i *_MIXED *T*_*lca *_&*T*_*j *_IS *T*_*lca*_)

inclTerms ← inclTerms ∪ *T*_*i*_

exclTerms ← exclTerms ∪ *T*_*lca*_

if (numInst(*T*_*k*_) < numInst(*T*_*i*_))

numInst(*T*_*k*_) = numInst(*T*_*i*_)

if (numInst(*T*_*lca*_) < numInst(*T*_*k*_))

numInst(*T*_*lca*_) = numInst(*T*_*k*_)

Since the relationship between *T*_*i *_and *T*_*j *_cannot be refined further than their relationship via *T*_*lca *_only *T*_*lca *_is assigned to *exclTerms*.

The second case occurs when *T*_*i *_MIXED *T*_*lca *_via *T*_*k *_and *T*_*j *_PART *T*_*lca*_. Since *T*_*j *_is part of *T*_*lca *_and *T*_*i *_is part of *T*_*k *_which is a kind of *T*_*lca *_then *T*_*j *_is a part of *T*_*k*_.

**8 **if (*T*_*i *_MIXED *T*_*lca *_&*T*_*j *_PART *T*_*lca*_)

inclTerms ← inclTerms ∪ *T*_*i*_

inclTerms ← inclTerms ∪ *T*_*j*_

exclTerms ← exclTerms ∪ *T*_*k*_

exclTerms ← exclTerms ∪ *T*_*lca*_

if (numInst(*T*_*k*_) < numInst(*T*_*i*_))

numInst(*T*_*k*_) = numInst(*T*_*i*_)

if (numInst(*T*_*k*_) < numInst(*T*_*j*_))

numInst(*T*_*k*_) = numInst(*T*_*j*_)

if (numInst(*T*_*lca*_) < numInst(*T*_*k*_))

numInst(*T*_*lca*_) = numInst(*T*_*k*_)

The final case occurs when both terms *T*_*i *_and *T*_*j *_are MIXED related to *T*_*lca *_via *T*_*k *_and *T*_*m *_respectively. What is common between both terms *T*_*i *_and *T*_*j *_is that they are both part of *T*_*lca*_. The number of instances associated with each term is adjusted to satisfy the partial order constraints associated with this case.

**9 **if (*T*_*i *_MIXED *T*_*lca *_&*T*_*j *_MIXED *T*_*lca*_)

inclTerms ← inclTerms ∪ *T*_*i*_

inclTerms ← inclTerms ∪ *T*_*j*_

exclTerms ← exclTerms ∪ *T*_*lca*_

if (numInst(*T*_*k*_) < numInst(*T*_*i*_))

numInst(*T*_*k*_) = numInst(*T*_*i*_)

if (numInst(*T*_*m*_) < numInst(*T*_*j*_))

numInst(*T*_*m*_) = numInst(*T*_*j*_)

if (numInst(*T*_*lca*_) < numInst(*T*_*k*_))

numInst(*T*_*lca*_) = numInst(*T*_*k*_)

if (numInst(*T*_*lca*_) < numInst(*T*_*m*_))

numInst(*T*_*lca*_) = numInst(*T*_*m*_)

After all terms have been compared with each other it is necessary to remove any terms from *inclTerms *that are found in *exclTerms*. This can occur when one comparison assigns a term to *inclTerms *while another comparison identifies the term as belonging to the excluded set. After all terms are compared each term in *inclTerms *should have the same number of instances associated with it. The number of instances that are associated with an annotation *G *is equal to the minimum number of instances that can be associated with any of the terms in *inclTerms *∩ *G*.

#### Finding the Nearest Common Annotation

Just as in semantic similarity of terms, where there is a common ancestor between two terms, there exists a nearest common annotation between two annotations. The concept of a nearest common annotation allows the extension of information based semantic similarity measures of terms, such as Resnik's and Lin's measures, to information based measures of semantic similarity of annotations.

We define the *nearest common annotation *(NCA) between two annotations *G*_1 _and *G*_2 _to be the annotation containing terms related to both annotations. The NCA should have the minimum possible number of instances associated with it such that either *G*_1 _or *G*_2 _can be derived from it. The set of terms *exclTerms *which results from applying SSA to two annotations *G*_1 _and *G*_2 _will return the set of terms associated with the NCA.

#### Measuring Similarity

By introducing the notion of nearest common annotation we can naturally extend Resnik's measure to measuring similarity of annotation. The LCA between two terms is replaced with the NCA of two annotations *G*_1 _and *G*_2_. Likewise, instead of applying *IC*_*Corpus *_(eqn. 1) to instances associated with a term we apply *IC*_*Corpus *_to instances of an annotation. Thus the extension of Resnik's measure from terms to annotations *G*_1 _and *G*_2_, *SSA*_*Resnik*_, becomes:

$$\begin{array}{c} \text{exclTerms}\leftarrow SSA(G_{1},G_{2})\\ SSA_{Resnik}(G_{1},G_{2})=\\ -\log\left(\frac{\underset{T_{i}\in exclTerms}{\min}numInst(T_{i})}{\max NumInst}\right) \end{array}$$

where *maxNumInst *is the number of distinct instances in the corpus.

Lin's measure may be extended as follows:

$$\begin{array}{c} \text{inclTerms}1\leftarrow SSA(G_{1},G_{1})\\ \text{inclTerms}2\leftarrow SSA(G_{2},G_{2})\\ icG1\leftarrow -\log\left(\frac{\underset{T_{i}\in inclTerms1}{\min}numInst(T_{i})}{maxNumInst}\right)\\ icG2\leftarrow -\log\left(\frac{\underset{T_{j}\in inclTerms2}{\min}numInst(T_{j})}{maxNumInst}\right)\\ SSA_{Lin}(G_{1},G_{2})=\frac{2*SSA_{Resnik}(G_{1},G_{2})}{icG1+icG2} \end{array}$$

In this case the SSA algorithm is used to find the non redundant terms that can be associated with an annotation.

#### Example

We compare the similarity of two gene product's annotations that returns a high measure of similarity when compared using our measure *SSA*_*Resnik*_. Two gene products, AAH1 and FUR1 whose annotations (listed in table 2) were taken from the SGD database [27] were compared producing a similarity value of 5.678. The number of instances associated with each term were obtained from the GOA [28]*s. cerevisiae *table of GO assignments.

**Table 2:** Example Annotations and Their Descriptions

Gene	Term	Description
AAH1	GO:0000034	adenine deaminase activity
	GO:0004000	adenosine deaminase activity
	GO:0005634	nucleus
	GO:0005737	cytoplasm
	GO:0006146	adenine catabolic process
	GO:0009117	nucleotide metabolic process
	GO:0009168	purine ribonucleoside monophosphate biosynthetic process
	GO:0016787	hydrolase activity
	GO:0019239	deaminase activity
	GO:0042254	ribosome biogenesis and assembly
	GO:0043101	purine salvage
	GO:0043103	hypoxanthine salvage

FUR1	GO:0004845	uracil phosphoribosyltransferase activity
	GO:0005622	intracellular
	GO:0008655	pyrimidine salvage
	GO:0009116	nucleoside metabolic process
	GO:0016740	transferase activity
	GO:0016757	transferase activity, transferring glycosyl groups

FUR1's annotation consisted of six terms: {GO:0004845, GO:0005622, GO:0008655, GO:0009116, GO:0016740, GO:0016757}. Each term's description is found in table 2. Likewise, AAH1's annotation consists of twelve terms: {GO:0000034, GO:0004000, GO:0005634, GO:0005737, GO:0006146, GO:0009117, GO:0009168, GO:0016787, GO:0019239, GO:0042254, GO:0043101, GO:0043103}. The NCA is constructed by applying the SSA algorithm to identify the set of contextual terms common to both annotations. Terms such as the root term 'all' are immediately added to *exclTerms*. The term 'cellular component' (GO:0005575) is added to *exclTerms *since another term 'cell part' is is_a related to it. The term 'nucleobase metabolic process' (GO:0009112) is a more specific type of 'nucloebase, nucleoside and nucleotide process' (GO:0055086) and the terms are added to *inclTerms *and *exclTerms *respectively. Similar assignments occur for 'nucleobase metabolic process' (GO:0009112)/'cellular metabolic process' (GO:0044237), 'nucleobase metabolic process' (GO:0009112)/'cellular process' (GO:0009987) as well as other terms.

The SSA algorithm return nine contextual terms, {'all' (all), 'cellular process' (GO:0009987), 'cellular metabolic process' (GO:0044237), 'nucleobase metabolic process' (GO:0009112), 'nucleobase, nucleoside, nucleotide and nucleic acid metabolic process' (GO:0006139), 'nucleobase, nucleoside and nucleotide metabolic process' (GO:0055086), 'cell part' (GO:0044464), 'intracellular' (GO:0005622), 'catalytic activity' (GO:0003824), 'metabolic compound salvage' (GO:0043094)}. The resulting annotation contains terms from all three ontologies in the GO. There are 19 instances associated with the annotation. The number of instances is determined by the most specific term: 'metabolic compound salvage' (GO:0043094). The total number of instances in the corpus is 5554. $SSA_{Resnick}=-\log\left(\frac{19}{5554}\right)\approx 5.678$. Since the highest value that *SSA*_*Resnik *_could return for the chosen corpus is ~8.622, taking the natural log of $\frac{1}{5554}$, 5.678 corresponds to high degree of similarity.

## Results

To validate our approach the discriminatory power of our method to identify clusters of related gene products was compared against Wang's measure of annotation similarity that also exploits the differences between types of relations. The average similarity of gene products found in the same biochemical pathway in the SGD database was compared against the average similarity of the same gene products compared with gene products found in other pathways. A large difference between these two values indicates the effectiveness of a similarity measure in discovering new pathways in a set of gene products. Average similarity of annotations inside and outside pathways was measured under four conditions: all terms; cellular component terms only; biological process terms only; and molecular function terms only.

A better test would be to take the average similarity of a set of gene products found in the same pathway and find the average or max of the average similarities of all other similarly sized sets of gene products. Of course this is intractable since the computational complexity of such a test is O(n!) since there are $\left(\begin{array}{c} N\\ n \end{array}\right)$ ways of creating a set of size n from a set of N elements.

**Figure 3:**
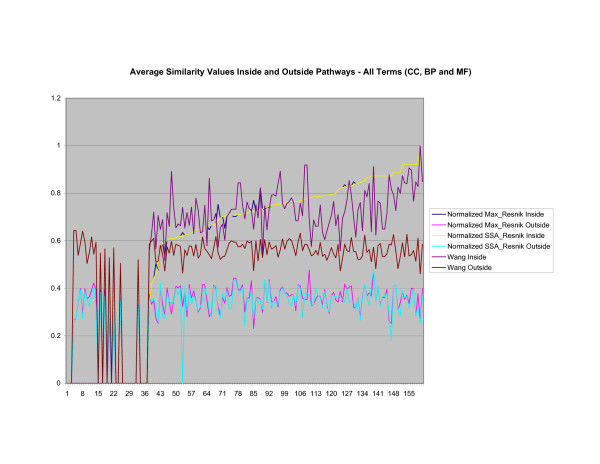
**Normalized *SSA*_*Resnik *_vs Wang's Method vs Normalized *Max*_*Resnik*_**. Values shown correspond to the average annotation similarity values between gene products with other gene products in the same pathway (taken from the SGD biochemical pathways database) and between gene products in a pathway with other gene products not found in the pathway.

**Figure 4:**
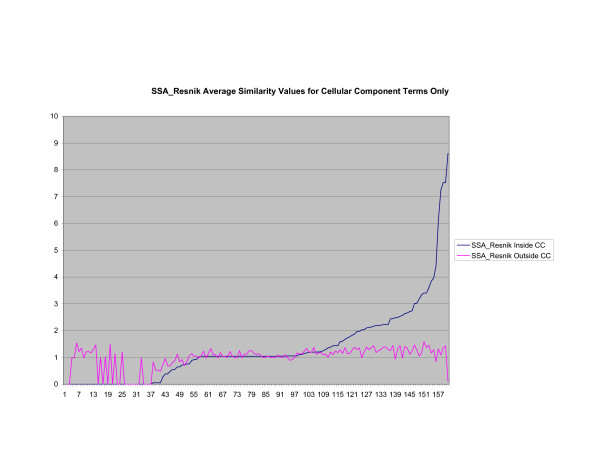
**Average Pathway Similarity Values of Annotations Consisting only of Cellular Component Terms Using *SSA*_*Resnik*_**. Average of *SSA*_*Resnik *_similarity values of gene products inside and outside a pathway.

**Figure 5:**
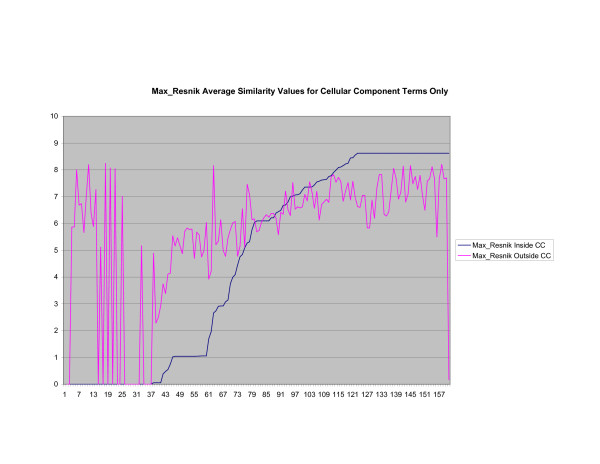
**Average Pathway Similarity Values of Annotations Consisting only of Cellular Component Terms Using *Max*_*Resnik*_**. Average of *Max*_*Resnik *_similarity values of gene products inside and outside a pathway.

**Figure 6:**
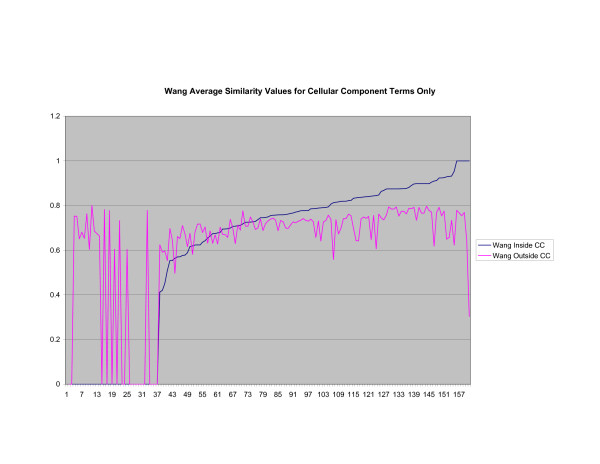
**Average Pathway Similarity Values of Annotations Consisting only of Cellular Component Terms Using Wang's Method**. Average of Wang's measure of similarity of gene products inside and outside a pathway.

**Figure 7:**
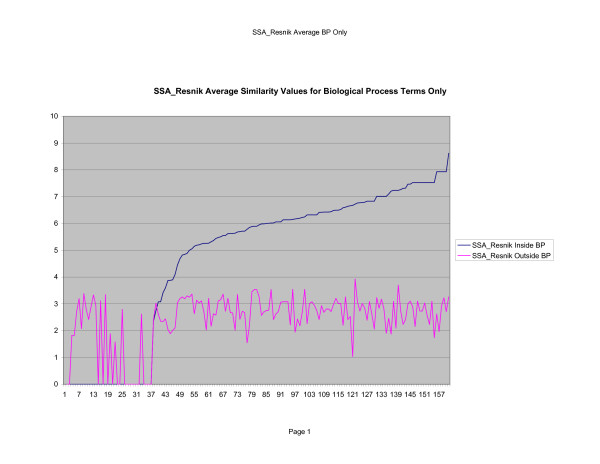
**Average Pathway Similarity Values of Annotations Consisting only of Biological Process Terms Using *SSA*_*Resnik*_**. Average of *SSA*_*Resnik *_similarity values of gene products inside and outside a pathway.

**Figure 8:**
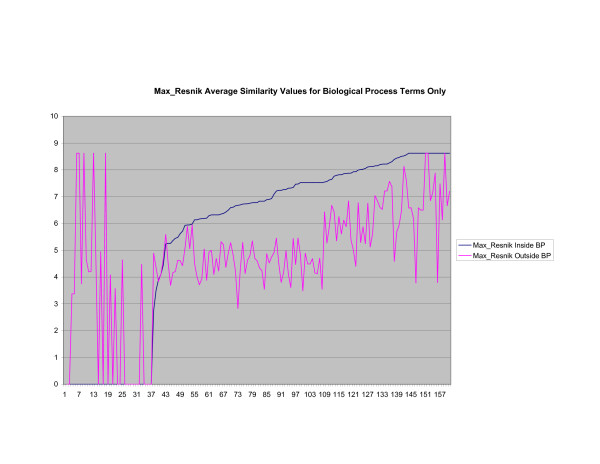
**Average Pathway Similarity Values of Annotations Consisting only of Biological Process Terms Using *Max*_*Resnik*_**. Average of *Max*_*Resnik *_similarity values of gene products inside and outside a pathway.

**Figure 9:**
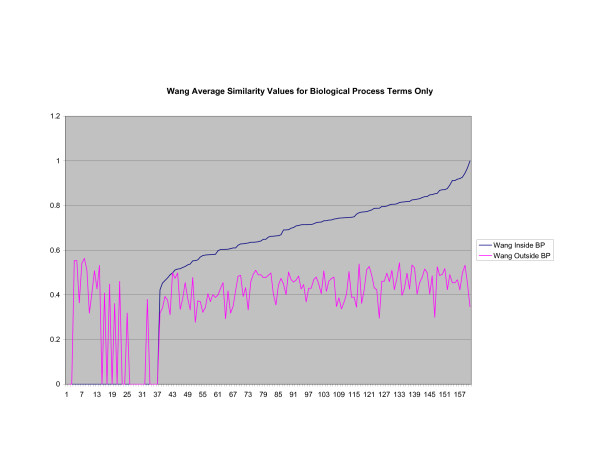
**Average Pathway Similarity Values of Annotations Consisting only of Biological Process Terms Using Wang's Method**. Average of Wang's measure of similarity of gene products inside and outside a pathway.

**Figure 10:**
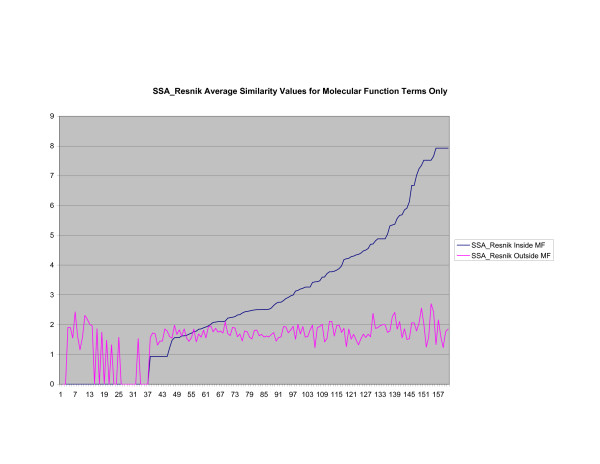
**Average Pathway Similarity Values of Annotations Consisting only of Molecular Function Terms Using *SSA*_*Resnik*_**. Average of *SSA*_*Resnik *_similarity values of gene products inside and outside a pathway.

**Figure 11:**
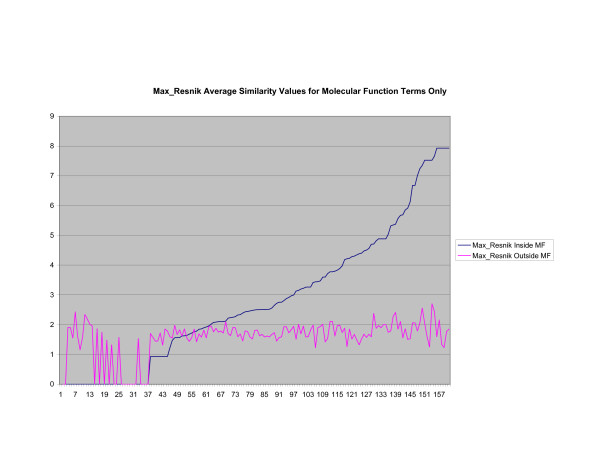
**Average Pathway Similarity Values of Annotations Consisting only of Molecular Function Terms Using *Max*_*Resnik*_**. Average of *Max*_*Resnik *_similarity values of gene products inside and outside a pathway.

**Figure 12:**
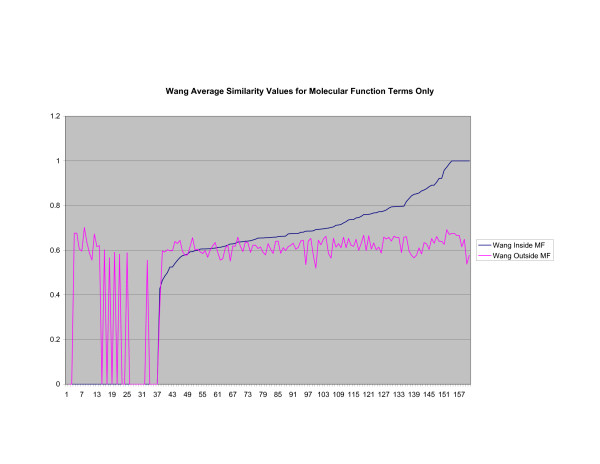
**Average Pathway Similarity Values of Annotations Consisting only of Molecular Function Terms Using Wang's Method**. Average of Wang's measure of similarity of gene products inside and outside a pathway.

**Figure 13:**
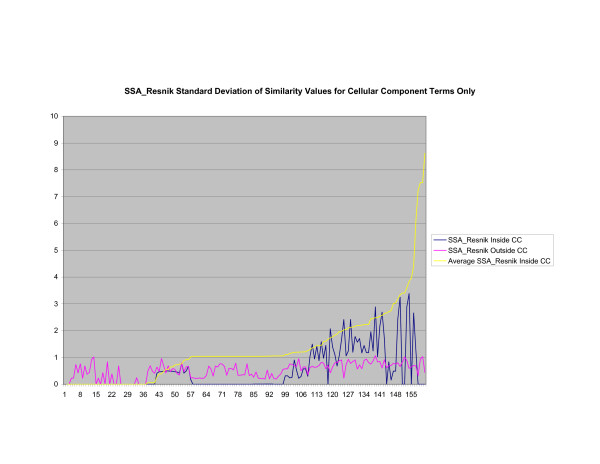
**Standard Deviation of Pathway Similarity Values of Annotations Consisting only of Cellular Component Terms Using *SSA*_*Resnik*_**. Standard deviation of *SSA*_*Resnik *_similarity values of gene products inside and outside a pathway.

**Figure 14:**
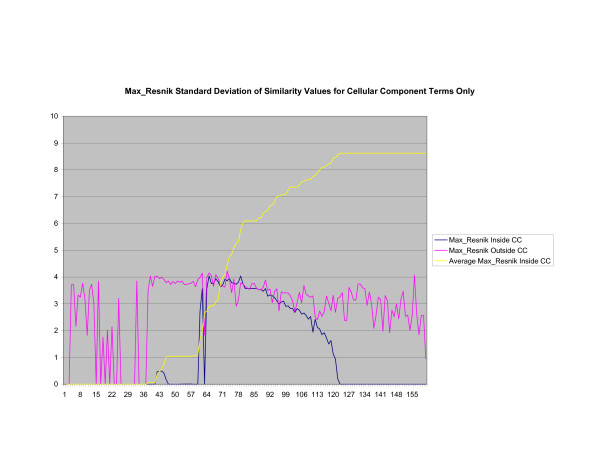
**Standard Deviation of Pathway Similarity Values of Annotations Consisting only of Cellular Component Terms Using *Max*_*Resnik*_**. Standard deviation of *Max*_*Resnik *_similarity values of gene products inside and outside a pathway.

**Figure 15:**
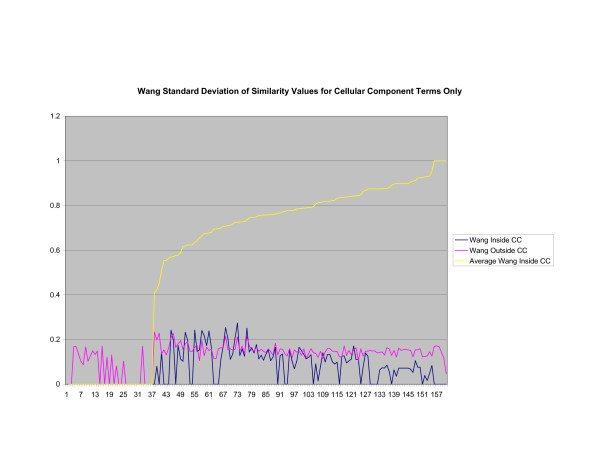
**Standard Deviation of Pathway Similarity Values of Annotations Consisting only of Cellular Component Terms Using Wang's Method**. Standard deviation of values of Wang's measure of similarity of gene products inside and outside a pathway.

**Figure 16:**
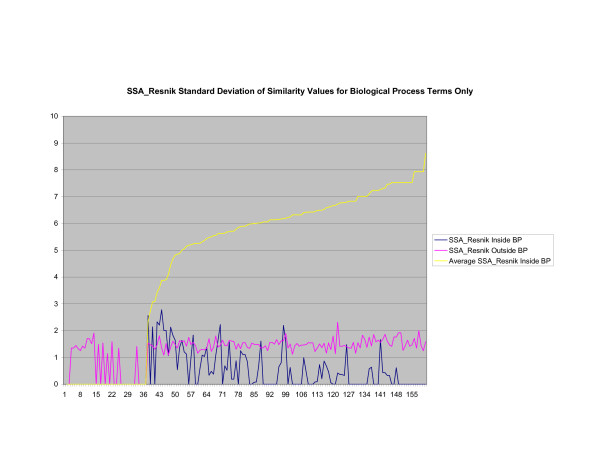
**Standard Deviation of Pathway Similarity Values of Annotations Consisting only of Biological Process Terms Using *SSA*_*Resnik*_**. Standard deviation of *SSA*_*Resnik *_similarity values of gene products inside and outside a pathway.

**Figure 17:**
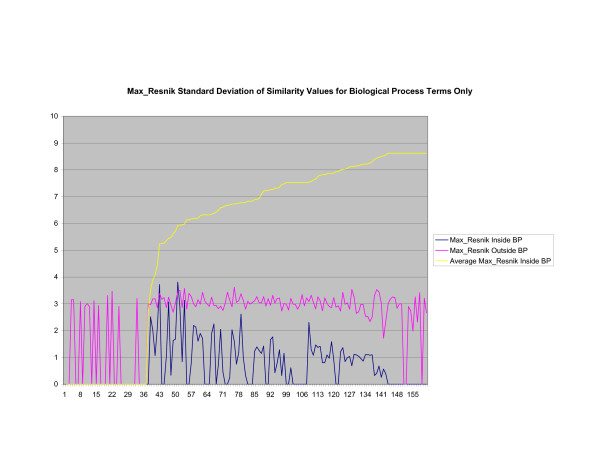
**Standard Deviation of Pathway Similarity Values of Annotations Consisting only of Biological Process Terms Using *Max*_*Resnik*_**. Standard deviation of *Max*_*Resnik *_similarity values of gene products inside and outside a pathway.

**Figure 18:**
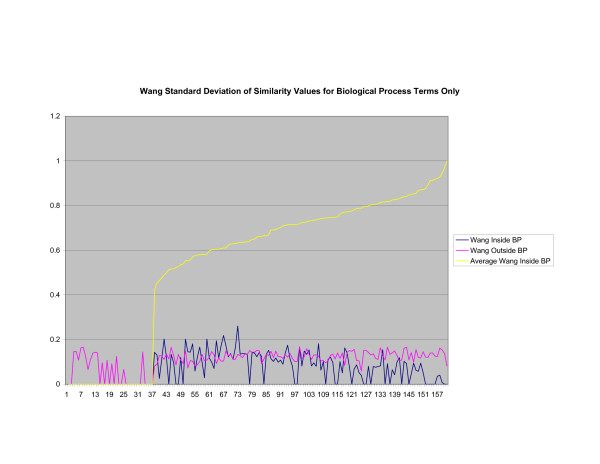
**Standard Deviation of Pathway Similarity Values of Annotations Consisting only of Biological Process Terms Using Wang's Method**. Standard deviation of values of Wang's measure of similarity of gene products inside and outside a pathway.

**Figure 19:**
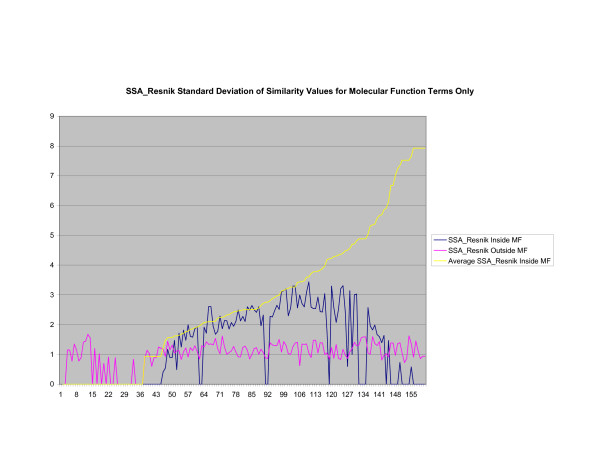
**Standard Deviation of Pathway Similarity Values of Annotations Consisting only of Molecular Function Terms Using *SSA*_*Resnik*_**. Standard deviation of *SSA*_*Resnik *_similarity values of gene products inside and outside a pathway.

**Figure 20:**
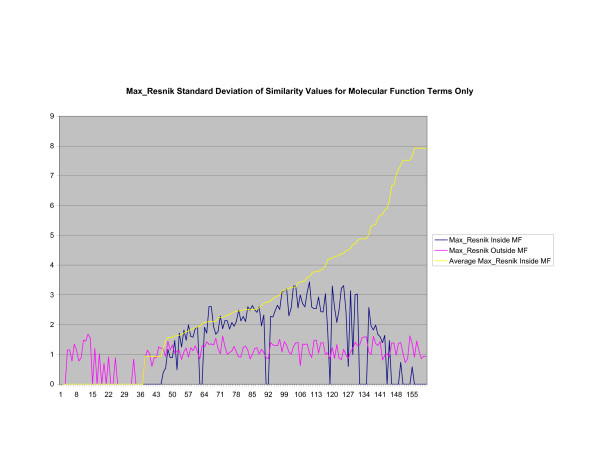
**Standard Deviation of Pathway Similarity Values of Annotations Consisting only of Molecular Function Terms Using *Max*_*Resnik*_**. Standard deviation of *Max*_*Resnik *_similarity values of gene products inside and outside a pathway.

**Figure 21:**
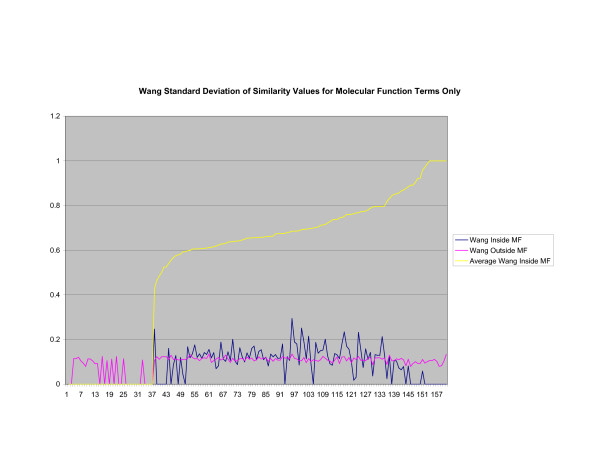
**Standard Deviation of Pathway Similarity Values of Annotations Consisting only of Molecular Function Terms Using Wang's Method**. Standard deviation of values of Wang's measure of similarity of gene products inside and outside a pathway.

Figure 3 show the results of a comparison of *SSA*_*Resnik *_with Wang's method and *M ax*_*Resnik *_on measuring the average annotation similarity, using all terms, of gene products inside and outside a pathway [data for figures 3, 4, 5, 6, 7, 8, 9, 10, 11, 12, 13, 14, 15, 16, 17, 18, 19, 20, 21 is found in Additional file 1]. The first 35 pathways are insufficiently annotated to produce meaningful results. Similarity values for *SSA*_*Resnik *_and *Max*_*Resnik *_were normalized to allow for direct comparison between similarity values. All measures behave similarly, the similarity values returned by Wang's method tends to increase as values returned by *SSA*_*Resnik *_increase. All measures tend to settle to an average similarity value when genes inside and outside a pathway are compared. Wang's method returns a higher value on average with values ranging between 0.5 and 0.6 as internal gene similarity increases. *SSA*_*Resnik *_and *Max*_*Resnik *_returns values between 0.3 and 0.4 for the average similarity value of genes inside a pathway with genes outside a pathway as similarity of genes within a pathway increases. If pathways are identified by the difference between the average similarity of gene products inside and outside a cluster then *SSA*_*Resnik *_and *Max*_*Resnik *_have greater discriminatory power. *SSA*_*Resnik *_and *Max*_*Resnik *_behave identically for most pathways when all terms are considered.

As shown in figures 4, 5, 6, when only terms from the cellular component sub-ontology are used the difference between *SSA*_*Resnik *_and *Max*_*Resnik *_becomes clear. *Max*_*Resnik *_returns a very high average similarity value between terms inside and outside a pathway. This may be an artifact of the low number of instances associated with cellular component terms. However when SSA is applied the average similarity values between annotations inside and outside pathways remains consistently low. *SSA*_*Resnik *_returns a comparatively high average similarity value for annotations inside pathways for approximately half the cases to which it can reasonably be applied. Wang's method behaves similarly to *Max*_*Resnik *_in this situation.

As shown in figures 7, 8, 9, if only biological process terms are used further dissimilarity between *Max*_*Resnik *_and *SSA*_*Resnik *_can be observed. The average similarity values of annotations inside a pathway with annotations outside a pathway is much higher for *Max*_*Resnik *_than for *SSA*_*Resnik*_. Wang's method and *SSA*_*Resnik *_behave similarly. Similarity values of annotations inside a pathway remain consistently higher than when the same annotations are compared with annotations outside the pathway for all methods.

The source of the similarity between *SSA*_*Resnik *_and *Max*_*Resnik *_can be identified when only molecular function terms are used, as shown in figures 10 and 11. In this case both methods behave exactly the same since there are no part of relations to exploit when comparing terms. Wang's method, shown in figure 12, returns a consistently high average similarity value for annotations inside a pathway compared with annotations outside a pathway.

Further discriminatory power can be achieved by considering the standard deviation of similarity values inside and outside a pathway. A set of gene products paired with other gene products in a pathway tend to have a high standard deviation of similarity values over all pairs mainly due to the small number of pairs being compared. Conversely, pairing gene products inside a pathway with those found outside the pathway should produce a set of similarity values with a lower standard deviation since annotations are expected to be dissimilar and values come from a larger set.

Figures 13, 14, 15 shows the standard deviation of similarity values of annotations consisting of cellular component terms inside pathways. *Max*_*Resnik *_returns a low internal standard deviation while reporting a consistently high standard deviation of similarity values when annotations inside a pathway are compared with annotations outside a pathway. The standard deviation of annotation similarity values between different pathways returned by both *SSA*_*Resnik *_and Wang's method are both consistently low. The standard deviation of all methods behave similarly as average similarity of annotations, consisting only of biological process terms, within pathways increase, as shown in figures 16, 17, 18. The same is also true of annotations consisting of molecular function terms, as shown in figures 19, 20, 21.

## Discussion and conclusion

The SSA algorithm provides the basis of a framework for extending instance based measures of term similarity to annotations. The algorithm's construction is based on the set of cases for how terms are related to each other when the ontology consists only of is_a and part_of relations. Due to the incomplete nature of the set of instances associated with a term it is necessary to adjust the number of instances associated with a term in order to satisfy the partial order constraints of each case fully. As the number of annotations of gene products increase and ontological terms are applied more consistently it may be possible to satisfy the constraints without such adjustment. Alternatively, the partial order constraints can be used to develop a similarity method which is less dependent on the set of instances associated with terms.

When terms from all three sub-ontologies (CC, BP and MF) are used similarity of annotations between *Max*_*Resnik *_and *SSA*_*Resnik *_are equivalent on proteins found in the SGD database. This is due to the high degree of specificity of molecular function terms, which are not related partonomically, which causes the two measures to return the same values. When only cellular component and biological process terms are used, based on the experimental evidence, *SSA*_*Resnik *_becomes a better identifier of proteins belonging to pathways. *SSA*_*Resnik *_may identify new gene products that belong to pathways but have a different molecular function to those proteins already identified as belonging to the pathway. Molecular function terms only play a small role in identifying new pathway proteins since proteins tend to have different molecular functions inside pathways.

By finding the set of instances that can be associated with an annotation it is possible to preserve, at the annotation level, the properties of instance based methods used to measure the similarity of terms. For two given annotations, the nearest common annotation (NCA) is a minimal set of terms such that either annotation could be derived from it. The SSA algorithm provides a method for finding the set of terms associated with the NCA.

By combining the SSA algorithm with Resnik's measure and the concept of nearest common annotation we have developed a measure that provides good discriminatory power to identify possible pathways and other functional groups from gene product annotations. More generally, the set of cases and their associated constraints further extend the set of principles that a reasonable measure of annotation similarity should be built on.

## Competing interests

The authors declare that they have no competing interests.

## Authors' contributions

BS proposed, designed and implemented the algorithm and table of constraints. BS wrote the manuscript. AQ and BG supervised and approved the production of this paper. SD contributed helpful suggestions for the final manuscript.

## Supplementary Material

Additional file 1 **Averages and Standard Deviations of Similarity Values. **Averages and standard deviations of similarity values of *Max*_*Resnik*_, *SSA*_*Resnik *_and Wang's method for each pathway in SGD. Click here for file

## References

[B1] Lord P, Stevens R, Brass A, Goble CA (2003). Semantic Similarity Measures as Tools for Exploring the Gene Ontology. Pacific Symposium on Biocomputing.

[B2] Lord P, Stevens R, Brass A, Goble C (2003). Investigating semantic similarity measures across the Gene Ontology: the relationship between sequence and annotation. Bioinformatics.

[B3] Sevilla J, Segura V, Podhorski A, Guruceaga JE Mato, Martinez-Cruz L, Corrales F, Rubio A (2005). Correlation between gene expression and GO semantic similarity. IEEE/ACM Transactions on Computational Biology and Bioinformatics.

[B4] Couto FM, Silva MJ, Coutinho PM (2007). Measuring semantic similarity between Gene Ontology terms, Data and Knowledge Engineering. Business Process Management – Where business processes and web services meet.

[B5] Lei Z, Dai Y (2006). Assessing protein similarity with Gene Ontology and its use in subnuclear localization prediction. BMC Bioinformatics.

[B6] Goodman N, Goodman N (1972). Seven strictures on similarity. Problems and Projects.

[B7] Arrell D (1987). What Goodman Should Have Said about Representation. The Journal of Aesthetics and Art Criticism Autumn.

[B8] Tversky A (1977). Features of Similarity. Psychological Rev.

[B9] Lin D (1998). An Information-Theoretic Definition of Similarity. Fifteenth International Conference on Machine Learning (ICML'98).

[B10] Popescu M, Keller J, Mitchell J (2006). Fuzzy Measures on the Gene Ontology for Gene Product Similarity. IEEEIACM Transactions on computational biology and bioinformatics.

[B11] Cross V Tversky's Parameterized Similarity Ratio Model: A Basis for Semantic Relatedness. Fuzzy Information Processing Society, 2006 NAFIPS 2006 Annual meeting of the North American.

[B12] Torsello A, Hidovic D, Pelillo M (2004). Four Metrics for Efficiently Comparing Attributed Trees. Proc of 17th International Conference on Pattern Recognition.

[B13] Guo X, Liu R, Shriver CD, Hu H, Liebman MN (2006). Assessing semantic similarity measures for the characterization of human regulatory pathways. Bioinformatics.

[B14] Wang JZZ, Du Z, Payattakool R, Yu PSS, Chen CFF (2007). A New Method to Measure the Semantic Similarity of GO Terms. Bioinformatics.

[B15] Pesquita C, Faria D, Bastos H, Falcao A, Couto F (2007). Evaluating GO-based Semantic Similarity Measures. BioOntologies SIG at ISMB/ECCB – 15th Annual International Conference on Intelligent Systems for Molecular Biology (ISMB).

[B16] Veale N , Seco JHT (2004). An Intrinsic Information Content Metric for Semantic Similarity in WordNet. ECAI 2004.

[B17] Schlicker A, Albrecht M (2007). FunSimMat: a comprehensive functional similarity database. Nucl Acids Res.

[B18] Rada R, Mili H, Bicknell E, Bletner M (1989). Development and Application of a Metric on Semantic Nets. IEEE Transactions on Systems, Man, and Cybernetics.

[B19] Lee JH, Kim MH, Lee YJ (1993). Information Retrieval Based on Conceptual Distance in IS-A Hierarchies. Journal of Documentation.

[B20] Wu Z, Palmer M (1994). Verb semantics and lexical selection. 32nd Annual Meeting of the Association for Computational Linguistics.

[B21] Resnik P (1995). Using Information Content to Evaluate Semantic Similarity in a Taxonomy. Proceedings of IJCAI-95.

[B22] Resnik P (1999). Semantic Similarity in a Taxonomy: An Information-Based Measure and its Application to Problems of Ambiguity in Natural Language. Journal of Artificial Intelligence Research.

[B23] Jiang J, Conrath D (1997). Semantic Similarity Based on Corpus Statistics and Lexical Taxonomy. Proc Int'l Conf Research in Computational Linguistics, ROCLING X.

[B24] Simon P (1987). Parts: a study in ontology.

[B25] Gene Ontology Consortium (2004). GO Editorial Style Guide.

[B26] Bittner T (2004). Axioms for parthood and containment relations in bio-ontologies. Unknown.

[B27] Cherry JM, Adler C, Ball C, Chervitz SA, Dwight SS, Hester ET, Jia Y, Juvik G, Roe T, Schroeder M, Weng S, Botstein D (1998). SGD: Saccharomyces Genome Database. Nucleic Acids Res.

[B28] Camon E, Magrane M, Barrell D, Lee V, Dimmer E, Maslen J, Binns D, Harte N, Lopez R, Apweiler R (2004). The Gene Ontology Annotation (GOA) Database: sharing knowledge in Uniprot with Gene Ontology. Nucleic Acids Research.

